# Supplementary material for: An open-source framework for large-scale, flexible evaluation of biomedical text mining systems
Source: J Biomed Discov Collab. 2008 Jan 29;3:1. doi: 10.1186/1747-5333-3-1 (PMC2276192; doi:10.1186/1747-5333-3-1)
Supplement: Additional file 1 — Evaluation platform source code. The additional file contains the software evaluation framework discussed in this paper. The most current release can be downloaded from SourceForge.net [1]. See the accompanying README file for instructions on installation and use. Also included in the distribution is a collection of UIMA wrappers for some commonly used BioNLP tools and annotated corpora. [file 1747-5333-3-1-S1.zip › bionlp_uima_repository-1.0.1/docs/api/allclasses-frame.html]

All Classes (Center for Computational Pharmacology Util/UIMA API)


**All Classes**
  

AbGene\_AE
  
AbGeneEISystem
  
ABNER\_AE
  
Abner\_Util
  
Annotation\_Util
  
AnnotationComparator
  
AnnotationComparator\_CC
  
AnnotationConsensusFilter\_AE
  
AnnotationFromFileLoader\_AE
  
AnnotationGroup
  
AnnotationGroupID
  
AnnotationMap
  
AnnotationPrinter
  
*AnnotationPrinterUtil*
  
AnnotationSet
  
AnnotationSetFilter\_AE
  
AnnotationSetOverrider\_AE
  
AnnotationString
  
AnnotationToFileOutput
  
Annotator
  
AnnotatorConverter\_AE
  
AnnotatorFilter\_AE
  
AnnotatorOverrider\_AE
  
Bio1CorpusParser
  
BioIECorpusParser
  
CCPAnnotation
  
CCPAnnotation\_Type
  
CCPAnnotationSet
  
CCPAnnotationSet\_Type
  
CCPAnnotator
  
CCPAnnotator\_Type
  
CCPClassMention
  
CCPClassMention\_Type
  
CCPClauseAnnotation
  
CCPClauseAnnotation\_Type
  
CCPComplexSlotMention
  
CCPComplexSlotMention\_Type
  
CCPDocumentAnnotation
  
CCPDocumentAnnotation\_Type
  
CCPDocumentInformation
  
CCPDocumentInformation\_Type
  
CCPDocumentSection
  
CCPDocumentSection\_Type
  
CCPDocumentSubSection
  
CCPDocumentSubSection\_Type
  
CCPMention
  
CCPMention\_Type
  
CCPNonComplexSlotMention
  
CCPNonComplexSlotMention\_Type
  
CCPOpenDMAP\_AE
  
CCPPhraseAnnotation
  
CCPPhraseAnnotation\_Type
  
CCPSemanticAnnotation
  
CCPSemanticAnnotation\_Type
  
CCPSentenceAnnotation
  
CCPSentenceAnnotation\_Type
  
CCPSlotMention
  
CCPSlotMention\_Type
  
CCPSpan
  
CCPSpan\_Type
  
CCPSyntacticAnnotation
  
CCPSyntacticAnnotation\_Type
  
CCPTextAnnotation
  
CCPTextAnnotation\_Type
  
CCPTokenAnnotation
  
CCPTokenAnnotation\_Type
  
CCPTypedDependency
  
CCPTypedDependency\_Type
  
ClassMention
  
*ClassMentionComparator*
  
ClassMentionConverter\_AE
  
ClassMentionRemovalFilter\_AE
  
ClassMentionTypeFilter\_AE
  
*ClassMentionTypes*
  
ComparatorConfigurator
  
ComparisonGroup
  
ComplexSlotMention
  
*ComplexSlotMentionComparator*
  
*ComplexSlotMentionTypes*
  
CorpusParser
  
DefaultPrintOutput
  
DMAPJcasTokenizer
  
DMAPStanfordPhraseGraph
  
DocumentLevelAnnotationCreator
  
DocumentLevelAnnotationCreator\_AE
  
DocumentSection
  
*DocumentSectionTypes*
  
DocumentUtil
  
*EIFileBasedSystem*
  
*EISystem*
  
EntityPartsAnnotationComparator
  
GenericDocument
  
GenericTextUtils
  
GeniaCorpusParser
  
GeniaTaggerOutputFileLoader\_AE
  
GeniaTaggerOutputFileParser
  
IdenticalClassMentionComparator
  
IdenticalComplexSlotMentionComparator
  
IdenticalMentionComparator
  
IdenticalSlotMentionComparator
  
IgnoreSpanComparator
  
InvalidSpanException
  
InvalidTypedDependencyException
  
IProLinkCorpus1Parser
  
IProLinkCorpus2Parser
  
IProLinkParser
  
KeX\_AE
  
KeXEISystem
  
KeXSentenceDetector\_AE
  
KeyValuePair
  
LingPipe\_Util
  
LingPipeEntityTagger\_AE
  
LingPipePOSTagger\_AE
  
LingPipeSentenceDetector\_AE
  
Mention
  
MentionComparator
  
MentionDocumentLevelSortComparator
  
MentionSortComparator
  
MutationFinder\_AE
  
NLPStats
  
OpenDmapAnnotator
  
OpenNLPSentenceDetector\_AE
  
OpenNLPSentenceSegmenter
  
OverlappingAnnotationResolver
  
OverlappingAnnotationResolver\_AE
  
PennBioTagger\_AE
  
PennBioTaggerEISystem
  
PennBioTokenizer\_AE
  
PennBioTokenizer\_Util
  
PorterStemmer\_AE
  
PorterStemmer\_Util
  
PRFResult
  
ProteinAnaphoraResolver
  
ReferenceProcessor
  
RunExecutable
  
SharedEndSpanComparator
  
SharedStartOrEndSpanComparator
  
SharedStartSpanComparator
  
SimpleCollectionReaderFormatOutput
  
SloppySpanComparator
  
SlotMention
  
*SlotMentionComparator*
  
*SlotMentionTypes*
  
SlotRemovalFilter\_AE
  
Span
  
SpanComparator
  
StanfordParser\_AE
  
StanfordParser\_Util
  
StrictSpanComparator
  
TestPrinter
  
TestSummarizer
  
TexasCorpusParser
  
TextAnnotation
  
*Tokenizer*
  
TypedDependencyCls
  
TypedDependencyCls\_Util
  
UIMA\_Annotation\_Util
  
UIMA\_Util
  
UIMASyntacticAnnotation\_Util
  
XMLEntityExtractor
  
XMLValidityChecker
  
YapexCorpusParser
